# Heterogeneity in the lymphocytic infiltration of deficient DNA mismatch repair colon cancers

**DOI:** 10.18632/oncotarget.26395

**Published:** 2018-12-04

**Authors:** Harry H. Yoon, Frank A. Sinicrope

**Affiliations:** Harry H. Yoon: Department of Medical Oncology, Mayo Clinic, Rochester, MN, USA; Frank A. Sinicrope: Department of Medical Oncology, Mayo Clinic, Rochester, MN, USA

**Keywords:** tumor infiltrating lymphocyte, colon stage III, mismatch repair, prognosis, heterogeneity

Colorectal cancer (CRC) consists of two major pathways resulting in a minority of tumors with deficient DNA mismatch repair (dMMR) and the majority with proficient mismatch repair (pMMR). In CRC, dMMR is most commonly due to sporadic epigenetic silencing of the *MLH1* MMR gene, or due to a germline mutation in an MMR gene (*MLH1, MSH2, MSH6, PMS2 or EpCAM*) conferring Lynch Syndrome with somatic inactivation of the second allele. Tumors with dMMR accumulate insertions and deletion mutations in DNA repeat sequences which manifest as microsatellite instability (MSI). In genes containing coding repeats, frameshift mutations are a source of immunogenic neo-antigens recognized by the immune system which can trigger lymphocytic infiltration within the tumor microenvironment. This pre-existing intratumoral T-cell response in dMMR tumors is believed to underlie their earlier stage at presentation with reduced propensity for metastasis and more favorable outcome compared to pMMR tumors.

**Figure 1:**
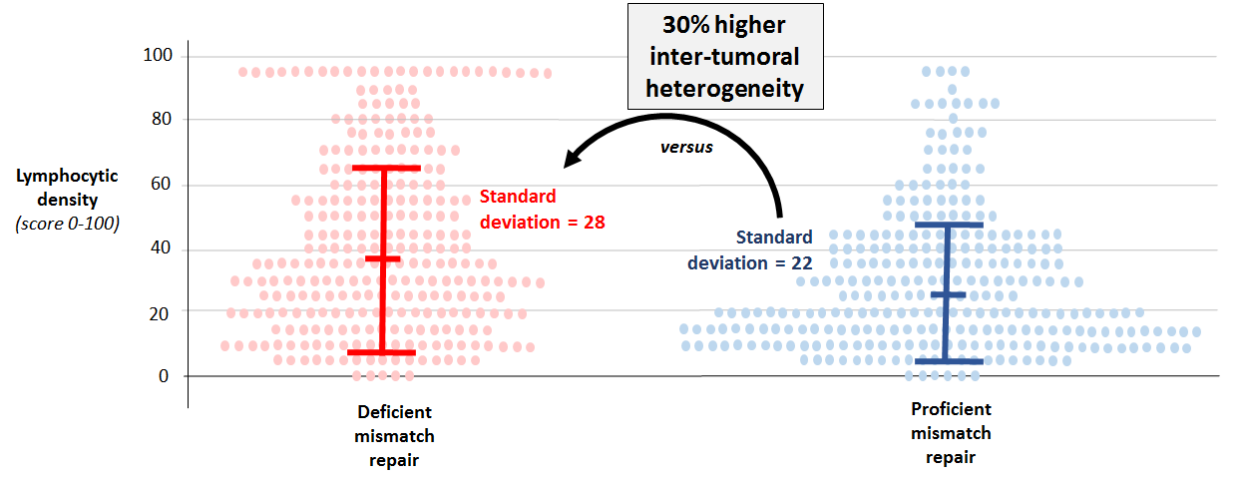
Density of lymphocytic infiltrates in tumor microenvironment exhibit greater inter-tumoral heterogeneity across mismatch repair-deficient (vs –proficient) colon cancers Data for CD3^+^ T cells at the invasive tumor margin are shown. Each dot represents one tumor. Bars show median +/- standard deviations. F-fold *P* value <.0001.

While it is believed that an abundant lymphocytic infiltrate is almost uniformly present in CRCs with dMMR, we determined the potential for inter-tumoral heterogeneity in lymphocytic infiltration among dMMR colon cancers. Specifically, we quantified the density of CD3+ and CD8+ T cells at the invasive margin (tumor’s leading edge) and tumor center in 561 stage III colon cancers. We examined all dMMR tumors and a randomly selected subgroup of pMMR tumors from patients who received adjuvant FOLFOX-based chemotherapy in a phase III clinical trial sponsored by the U.S. National Cancer Institute (N0147) [1]. CD3+ is a pan-T cell marker and CD8+ is a marker of cytotoxic T cells. Consistent with prior data, we found that CD3+ and CD8+ T-cell densities in the tumor microenvironment were higher in dMMR vs pMMR tumors overall. However, we unexpectedly observed that dMMR colon cancers demonstrated significantly greater inter-tumoral heterogeneity in the density of their lymphocytic infiltrate compared to pMMR tumors (Figure 1). Inter-tumoral heterogeneity in densities was significantly increased by 30-88% among dMMR vs pMMR cancers (P < .0001 for all four T-cell subtypes [CD3+ or CD8+ at the invasive margin or tumor center]). Importantly, a substantial proportion of dMMR tumors (26% to 35% depending on T-cell subtype) exhibited T-cell densities as low as those found in the bottom half of densities within pMMR tumors. Stated differently, approximately one-third of dMMR colon cancers do not have a rich lymphocyte infiltrate. A number of factors could underlie the inter-tumoral heterogeneity among dMMR tumors including differences in the overall mutation burden, unstable microsatellites, or variability in neoantigen profiles [2]. In a recent study, dMMR CRCs from patients with Lynch Syndrome had a higher density of intratumoral CD3+ T cells (vs sporadic dMMR) in association with increased somatic mutations and higher neoantigen burden [3].

Inter-tumoral heterogeneity in T-cell densities has implications for prognosis in both pMMR and dMMR groups. We found that patients whose tumors exhibited a low T-cell density (regardless of T-cell subtype or intratumor location) had a shorter overall survival compared to those with tumors exhibited a high T-cell density. CD3+ T-cell density at the invasive margin was prognostically strongest, and other T-cell subtypes did not add further prognostic value. Patients whose tumors had a low CD3+ T-cell density at the invasive margin experienced a 4-fold worsening of overall survival compared to patients whose tumors had a high CD3+ density. Importantly, the prognostic impact of CD3+ density remained statistically significant after adjustment for multiple confounders, including T and N stage, tumor grade, tumor sidedness, *BRAF/KRAS* mutation status, smoking and age.

A subset of pMMR tumors were found to exhibit a rich T-cell infiltrate, and patients with pMMR tumors exhibiting a low T-cell infiltrate had shorter OS, independent of confounders as previously shown by ourselves [4] and others [5]. Analysis of specimens from TCGA revealed that ∼5% of pMMR colon carcinomas are hypermutated using the cutoff of >12 mutations per 106 bases [6]. Mutations in the *POLE* proofreading (exonuclease) domain have been detected in 1–2% of CRCs and found to be associated with ultra-hypermutation and pMMR status. *POLE* encodes the catalytic subunit of DNA polymeraseε, which replicates the leading DNA strand before cell division. Recent sequencing data of nearly 80,000 tumors from adult patients found that MSI-high was mostly restricted to tumors in the 10-100 mutations/Mb range, whereas tumors with >100 mutations/Mb (ultra-hypermutated) were microsatellite stable and enriched for replicative polymerase mutations [7]. *POLE* mutations in patients with stage II-IIII CRCs were associated with increased intratumoral CD8+ T-cell infiltration, similar in extent to that observed in dMMR tumors, and with a significantly reduced risk of recurrence compared with patients with pMMR CRCs [8].

An important implication of our data that warrants further study is whether the inter-tumoral heterogeneity of lymphocytic infiltrates across dMMR tumors can predict the efficacy of immunotherapy. Data from melanoma patients suggest that the presence of a rich intratumoral CD8+ T-cell infiltrate may predict therapeutic benefit from programmed cell death receptor-1 (PD-1) blockade [9]. A major advance in cancer therapy was the finding that solid tumors with dMMR/MSI-high experience durable responses after treatment with PD-1 blockade, leading to regulatory approval for anti-PD-1 therapy in solid tumors with dMMR or MSI-high after progression on standard therapy [10]. However, anti-PD-1 antibody monotherapy yields response rates of only 31-36% in patients with advanced dMMR CRCs after failure of conventional therapy [11]. An ongoing adjuvant phase III study (ATOMIC) is evaluating whether the addition of the anti-PD-L1 antibody, atezolizumab, to standard chemotherapy can improve survival in patients with resected, stage III dMMR colon cancers. In this study, the predictive utility of lymphocytic density will be examined in relationship to patient outcome. In contrast to advanced dMMR tumors, anti-PD-1 therapy has not shown benefit for unselected patients with pMMR or microsatellite stable CRCs. It remains unknown if a subset of highly immunoreactive pMMR tumors may benefit from immunotherapy.
